# Interleukin-1β triggers matrix metalloprotease-3 expression through p65/RelA activation in melanoma cells

**DOI:** 10.1371/journal.pone.0278220

**Published:** 2022-11-29

**Authors:** Junichi Nunomura, Rei Nakano, Atsuto Naruke, Yoko Suwabe, Masumi Nakano, Naoya Yachiku, Manami Kuji, Mana Sugimura, Shinichi Namba, Taku Kitanaka, Nanako Kitanaka, Hiroshi Sugiya, Tomohiro Nakayama

**Affiliations:** 1 Laboratories of Veterinary Radiotherapy, Nihon University College of Bioresource Sciences, Fujisawa, Kanagawa, Japan; 2 Laboratory for Mucosal Immunity, Center for Integrative Medical Sciences, RIKEN Yokohama Institute, Yokohama, Kanagawa, Japan; 3 Japan Animal Specialty Medical Institute, Tsuzuki, Yokohama, Kanagawa, Japan; University of Cincinnati, UNITED STATES

## Abstract

Melanoma shows highly aggressive behavior (i.e., local invasion and metastasis). Matrix metalloprotease-3 (MMP-3), a zinc-dependent endopeptidase, degrades several extracellular substrates and contributes to local invasion by creating a microenvironment suitable for tumor development. Here, we report that interleukin-1β (IL-1β) triggers the MMP-3 expression in canine melanoma cells. The activity of MMP-3 in the culture supernatant was increased in IL-1β-treated melanoma cells. IL-1β time- and dose-dependently provoked the mRNA expression of MMP-3. IL-1β induced the migration of melanoma cells; however, this migration was attenuated by UK356618, an MMP-3 inhibitor. When the cells were treated with the nuclear factor-κB (NF-κB) inhibitor TPCA-1, the inhibition of MMP-3 expression was observed. In IL-1β-treated cells, the phosphorylation both of p65/RelA and p105 was detected, indicating NF-κB pathway activation. In p65/RelA-depleted melanoma cells, IL-1β-mediated mRNA expression of MMP-3 was inhibited, whereas this reduction was not observed in p105-depleted cells. These findings suggest that MMP-3 expression in melanoma cells is regulated through IL-1β-mediated p65/RelA activation, which is involved in melanoma cell migration.

## Introduction

The tumor microenvironment (TME) influences tumor development, such as its initiation, promotion, growth, and metastasis [1–7]. Several inflammatory and microenvironmental factors, including cytokines, chemokines, and growth factors, produced by the stroma, tumor-infiltrating leukocytes, or the cancer cells themselves, directly or indirectly contribute to the creation of TME.

Interleukin-1 (IL-1) is a mediator of immune response and is also known to be a pro-inflammatory cytokine [8]. Upregulation of IL-1, including IL-1β, in TME of various tumor types has been reported, which is thought to promote tumor malignancy by inducing the expression of other pro-inflammatory genes [9–11].

Matrix metalloproteases (MMPs) are a family of zinc-dependent endopeptidases known for their ability to degrade components of the extracellular matrix (ECM), such as collagen, laminin, elastin, fibronectin, and proteoglycans [12]. MMPs are divided into five subclasses by substrate specificity and subcellular distribution: collagenases, gelatinases, stromelysins, matrilysins, and membrane-type MMPs [13]. MMPs play crucial roles in cancer progression by modulating TME, modulating cell growth, and serving as a key protein in tumor progression [14].

MMP-3, also referred to as stromelysin-1, is a proteinase that degrades a broad array of extracellular substrates, such as type IV, V, IX, and X collagens, laminins, elastin, fibronectin, and the non-fibrillar collagens [15, 16]. MMP-3 is involved in activating other pro-MMPs, such as MMP-1, MMP-8, MMP-9, and MMP-13 [16–19]. In addition, MMP-3 is associated with cancer progression as its upregulation is reported in different cancers, such as advanced urothelial carcinoma [20], small cell lung carcinoma [21], and high-grade endometrial sarcoma [22]. A high expression of MMP-3 was reported to correlate with the tumor size of the melanoma in the patient, suggesting the contribution of MMP-3 to the processes is involved in the invasiveness of malignant melanomas [23, 24].

The nuclear factor of the κ-light chain of enhancer-activated B cells (NF-κB) is a family of nuclear transcription factors tightly correlated with human diseases, such as inflammation and cancer [25–29]. The NF-κB family comprises five members: p65/RelA, RelB, c-Rel, p50/p105 (NF-κB1), and p52/p100 (NF-κB2). In NF-κB1 and NF-κB2, p50 and p52 are generated by proteolytical processing from the precursor form, p105, and p100, respectively. The NF-κB family of proteins share the Rel homology domain at their N-termini, which acts as a DNA-binding/dimerization domain [25, 29]. In resting cells, the NF-κB dimeric complexes are sequestered as an inactive form in the cytoplasm, which directly interacts with inhibitory proteins, known as IκB family proteins. In cells stimulated with various stimuli, the interaction between IκB and NF-κB is disrupted, provoking the NF-κB activation. The NF-κB dimeric complexes are released and subsequently translocated into the nucleus, which triggers the induction of different genes by binding to the promoter elements [25, 29]. The NF-κB signaling pathway comprises two pathways, canonical and non-canonical pathways. Pro-inflammatory cytokines mediate the canonical NF-κB signaling pathway activation, whereas other ligands mediate the activation of the non-canonical NF-κB signaling pathway [29–31].

Melanoma is an aggressive tumor that spontaneously occurs in dogs [32]. Most cases of melanoma in dogs are malignant. The feature of canine malignant melanoma (*i*.*e*., rapid metastasis and resistant to chemotherapy) is associated with a worse prognosis, mimicking human mucosal melanomas [33–36]. Canine melanoma shares several clinical, biological, and genetic features with its human counterpart. Therefore, canine melanoma has been recognized as a suited model for studying human melanoma [33–38].

In the present study, we demonstrate that the canonical NF-κB signaling pathway, NF-κB p65/RelA activation, is involved in the IL-1β mediated-MMP-3 expression in canine melanoma cells.

## Materials and methods

### Cell culture

Canine melanoma cells (MCM-N1 cell line; 13-year-old male dog; chromosome number, 2n = 74) obtained from DS Pharma Biomedical Co., Ltd. (Osaka, Japan). The other canine melanoma cell lines (CMe1, CMMe2 and LMe) were kindly provided by Dr. Takayuki Nakagawa (Laboratory of Veterinary Surgery, Graduate School of Agricultural and Life Sciences, The University of Tokyo) [39–41]. The cells were cultured in 75-cm^2^ culture flasks containing DMEM-LG (FUJIFILM Wako Chemical Corp., Osaka, Japan) supplemented with 10% FBS, 100 unit/mL penicillin and 100 μg/mL streptomycin, and maintained at 37°C in the environment of a humidified incubator with 5% CO_2_ as described previously [42, 43]. The cells were treated with recombinant canine IL-1β (Kingfisher Biotech, Inc., Saint Paul, MN, USA) for the indicated periods and concentrations. For inhibitor treatment, the cells were pretreated with 2-((aminocarbonyl)amino)-5-(4-fluorophenyl)-3-thiophenecarboxamide (TPCA-1, 10 μM for 1 h, MedChemExpress, Monmouth Junction, NJ) or UK356618 (50 nM for 2 h, MedChemExpress, Monmouth Junction, NJ), then the cells were treated with recombinant canine IL-1β.

### Real-time polymerase chain reaction (RT-PCR)

RT-qPCR was conducted as previously described [42–53]. The samples of canine melanoma cells were collected with TRIzol reagent (Life Technologies, Carlsbad, CA). First-strand cDNA synthesis from 500 ng of the extracted total RNA was performed with PrimeScript RT Master Mix (TaKaRa Bio, Inc., Shiga, Japan). Real-time RT-PCR was carried out in a total reaction volume of 25 μL containing 2 μL of the first-strand cDNA, 12.5 μL SYBR Premix Ex Taq II (TaKaRa Bio, Inc.), and 0.4 μM forward and reverse primers specific for canine MMP-3 and NHE1. Primers of the TATA box binding protein (TBP), a housekeeping protein, were used as a control. Table 1 shows the primer sequences used. PCR was performed with the following cycling conditions using the Thermal Cycler Dice Real-Time System II (TaKaRa Bio, Inc.): one cycle of denaturation at 95°C for 30 sec, 40 cycles of denaturation at 95°C for 5 sec, and annealing/extension at 60°C for 30 sec. The results were analyzed using real-time RT-PCR software using the second derivative maximum method and the comparative cycle threshold (ΔΔCt) method. Amplification of TBP from the same amount of cDNA and the amplification of the cDNA from canine melanoma cells at time 0 were used as a reference and a calibration standard, respectively.

**Table 1:** Primer sequences for RT-qPCR.

Gene Name	Gene bank ID	Primer sequences
*MMP-3*	NM_001002967.1	F: 5ʹ- TGACGATGATGAACAATGGACAAG-3ʹ
		R: 5ʹ- GCTAGGGTCAGCCGAGTGAAAG-3ʹ
*SLC9A1 (NHE1)*	NM_001287028.2	F: 5’- CCCAGGATTGTGCAATAGTCAGAG-3’
		R: 5’-GGTGGCTTTGAACATGGTTGTC-3’
*TBP*	XM_863452	F: 5’-ACTGTTGGTGGGTCAGCACAAG-3’
		R: 5’-ATGGTGTGTACGGGAGCCAAG-3’

### Western blotting

Protein separation and Western blotting were performed as previously described [42–53]. Canine melanoma cells were lysed with 20 mM HEPES buffer (pH 7.4, FUJIFILM Wako Chemical Corp.) containing a complete mini EDTA-free protease inhibitor cocktail (Roche, Mannheim, Germany), 1 mM PMSF (FUJIFILM Wako Chemical Corp.), and 10 mM sodium fluoride (FUJIFILM Wako Chemical Corp.). The Bradford method determined protein concentrations of cell lysates [54]. After denaturing by boiling at 95°C for 5 min in SDS buffer (TaKaRa Bio, Inc.), proteins were electrophoretically separated on 7.5% or 12% Mini-PROTEAN TGX gel (Bio-Rad, Hercules, CA). The separated proteins were subsequently transferred to PVDF membranes (Bio-Rad). After blocking with Block Ace (KAC Co., Ltd., Hyogo, Japan) at room temperature for 50 min, the PVDF membranes were incubated at room temperature for 120 min with the following primary antibodies: t-p65/RelA (Cell Signaling Technology Japan, K.K., Tokyo, Japan, clone: D14E12, 1:1000), p-p65/RelA (Cell Signaling Technology Japan, K.K., clone: 93H1, 1:1000), t-p105 (Cell Signaling Technology Japan, K.K., clone: D4P4D, 1:1000), p-p105 (Cell Signaling Technology Japan, K.K., clone: 18E6, 1:1000), and β-actin (Sigma-Aldrich Inc., St Louis, MO, clone: AC74, 1:10,000). Then, the membranes were washed and incubated with an HRP-conjugated anti-rabbit or anti-mouse IgG antibody (Sigma-Aldrich Inc., 1:10000) at room temperature for 90 min. Immunoreactivity was detected using ECL Western Blotting Analysis System (Cytiva Japan, Tokyo, Japan), and the chemiluminescent signals were detected and analyzed using Amersham 800 (Cytiva Japan).

### Transfection of siRNA

The lipofection of siRNA was conducted as previously described [42–53]. Canine melanoma cells were seeded in a 35 mm or a 90 mm dish at a density of 1 × 10^5^ or 5 × 10^5^ cells, respectively. To transfect siRNA, the cells were incubated in Opti-MEM (Life Technologies) containing 5 μL/mL Lipofectamine 2000 (Life Technologies) and 100 nM p65/RelA, p105, or scramble siRNA for 6 h. After transfection, the medium was changed to DMEM-LG with 10% FBS, and cultures were retained at 37°C in a humidified incubator at 5% CO_2_ for 5 d. Table 2 shows the siRNA sequences. The efficiency of the siRNAs was checked by Western blotting.

**Table 2:** Sequences for siRNA transfection.

Gene Name	GenBank ID	siRNA sequences
*p65/RelA*	XM_014121307.2	GCAUCUCCCUGGUCACCAA
*p105*	AB183419.1	CUGCAAAGGUUAUUGUUCA

### MMP-3 activity assay

Canine melanoma cells were seeded in 6-well culture plates at a density of 3.0 × 10^5^ cells/well [55]. The cells were starved for 24 h and subsequently stimulated with IL-1β for 0 to 48 h. After stimulation, the culture medium was collected. MMP-3 activity in the culture medium was assayed using a chromogenic substrate containing an MMP3 Inhibitor Screening Assay kit (Abcam, Cambridge, UK) according to the manufacturer’s instructions.

### Migration assay

As described previously, a migration assay determined canine melanoma cell migration [55]. The Culture-Insert 2 Well (ibidi GmbH, Am Klopferspitz, Germany) was placed on the 35 mm dish, and 70 μL of cell suspension at a 1×10^6^/mL was dispensed in each well of the Culture-Insert 2 Well. After incubation at 37°C for 24 h, the cell culture inserts were carefully removed. Then IL-1β was applied for 0–24 h. Analysis of phase-contrast images was performed using the MRI Wound Healing Tool for ImageJ [56].

### Invasion assay

The invasion of canine melanoma cells was assayed using a CytoSelect 24-Well Cell Invasion Assay (8.0 μm pore diameter, basement membrane, colorimetric format, Cell Biolabs Inc., San Diego, CA) according to the manufacturer’s instructions.

### Adhesion assay

Matrigel (Corning, Glendale, AZ) was reconstituted with DEME-LG at a concentration of 40 μg/mL. A 96-well plate was coated with reconstituted Matrigel at 2 μg/50 μL/well and incubated for 18 h at 25°C. After washing the plate with PBS, canine melanoma cells were seeded at 3,000 cells/200 μL in each well of a 96-well plate and incubated for 60 min at 37°C, 5% CO_2_. Unattached cells were washed and discarded by PBS. The attached cells were stained by MTT assay [43]. MTT assay reagent (Dojindo, Tokyo, Japan) was dissolved in PBS at a concentration of 5 mg/mL, and 20 μL of the reagent was added to each well for 1 h with 5% CO_2_ at 37°C. Following incubation, PBS (100 μL) was added to each well. After 1 min, the supernatant was discarded, and the MTT formazan crystals were dissolved in 200 μL of 0.04 M hydrochloric acid (HCl) in 2-propanol. The optical density (O.D.) was detected by a microplate reader (Fluoroskan Ascent FL, Thermo Fisher Scientific K.K., Kanagawa, Japan) at 570 nm wavelength.

### Intracellular pH assay

The cells were seeded on 35 mm glass base dishes at a density of 4,000 cells/cm^2^. The cells were incubated with 5 μM BCECF-AM for 30 min at 37°C in the dark. Following incubation, the cells were washed twice with PBS. After washing, the culture medium was replaced with imaging buffer (containing 120 mM NaCl, 5 mM KCl, 0.96 mM NaH_2_PO_4_, 1 mM MgCl_2_, 11.1 mM glucose, 1 mM CaCl_2_, 1 mg/mL bovine serum albumin and 10 mM HEPES; pH 7.4). The glass base dishes with fluorescent dye-loaded cells were placed at room temperature on the stage of a confocal laser scanning microscope (LSM510). The data were collected from 20 cells, randomly selected ×20 fields from triplicate samples. The fluorescence intensity values were converted to absolute pH using the high K^+^/nigericin calibration method (cells on the same microplate were exposed to HEPES buffer pH 8.1, 7.4, and 6.2 in the presence of 140 mM KCl and 10 μg/ml nigericin.

### Statistical analysis

Statistical analyses were performed using StatMate IV, and data from all experiments are presented as the mean ± standard error. Data from the time-course study and other experiments were analyzed using two-way analysis of variance (ANOVA) and one-way ANOVA, respectively. Tukey’s test was used as a post-hoc analysis. *P*-values less than 0.05 were considered statistically significant.

## Results

### IL-1β induces the expression and release of MMP-3 in melanoma cells

**Fig 1:**
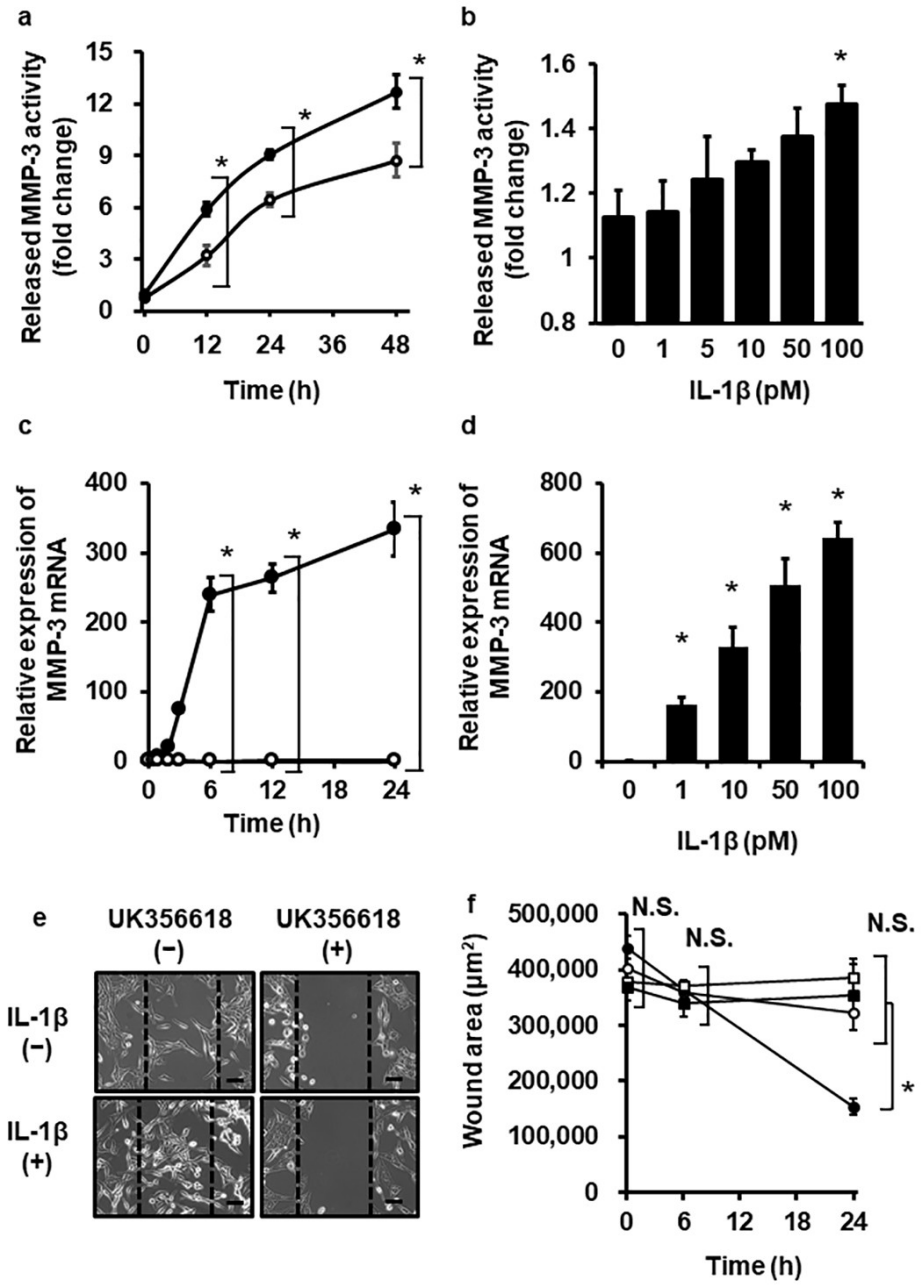
Expression of MMP-3 and cellular migration in IL-1β-treated canine melanoma cells. MMP-3 activity in the culture medium (a) and MMP-3 mRNA expression (c) of canine melanoma cells in the presence (closed circle) or absence (open circle) of canine recombinant IL-1β (100 pM). MMP-3 activity in the culture medium (b) and MMP-3 mRNA expression (d) in melanoma cells incubated with IL-1β (0 to 100 pM) for 24 h. TBP was used as a reference. Relative mRNA expression of MMP-3 in IL-1β-treated melanoma cells was compared with the expression at 0 h. (e, f) The cells were treated with (closed) or without (open) 100 pM IL-1β for 24 h in the presence (square) or absence (circle) of UK356618 (50 nM), an MMP-3 inhibitor, for 2 h. The representative data of cell migration (e) and wound area (f) values are shown. The data are represented as the mean ± standard error (SE) of three biological replicates. *P<0.05.

When canine melanoma cells were stimulated with IL-1β at the concentration of 100 pM for 0–48 h, the activity of MMP-3 in the culture media supernatant was increased (Fig 1a), which implies IL-1β-stimulated MMP-3 release. We observed that a dose-dependent upregulation of MMP-3 release in culture media of melanoma cells stimulated with IL-1β at the range of 0 to 100 pM for 24 h (Fig 1b). In canine melanoma cells stimulated with IL-1β, MMP-3 mRNA expression was enhanced time- and dose-dependently, as depicted in Fig 1c and 1d, respectively. To investigate the effect of IL-1β on pH in TME for the activity of MMP-3 via Na^+^/H^+^-exchanger NHE1, we checked the changes in intracellular pH using BECEF and the mRNA expression of NHE1 in IL-1β-treated cells. As shown in S1a and S1b Fig, IL-1β showed no significant difference in intracellular pH and the mRNA expression of NHE1. Thus, IL-1β mediates the MMP-3 expression and release in canine melanoma cells.

### MMP-3 contributes to the migration of melanoma cells

Cellular invasion, adhesion, and migration are pivotal steps in tumor progression. To investigate the role of MMP-3, the inhibitor for MMP-3 protease activity UK356618 was applied to the melanoma cells, and cellular invasion, adhesion, and migration mediated by IL-1β were determined. As Fig 1e and 1f summarize, IL-1β stimulated melanoma cell migration. However, in melanoma cells treated with UK356618 (50 nM), IL-1β failed to mediate cell migration, implying that MMP-3 is involved in IL-1β-induced melanoma cell migration. We performed a cellular invasion assay using a Boyden chamber coated with basement membrane extract. However, we could not detect cellular invasion in melanoma cells (S2a Fig). We also checked cellular adhesion using a Matrigel-coated dish. Cellular adhesion of IL-1β, UK356618, and UK356618+ IL-1β-treated cells showed no significant difference compared to control (S2b Fig). In the previous study, IL-1β did not affect cellular proliferation for 0 to 48 h [42]. Taken together, MMP-3 plays an important role in the cellular migration of IL-1β-treated melanoma cells via protease activity.

### Involvement of NF-κB in IL-1β-induced MMP-3 expression

**Fig 2:**
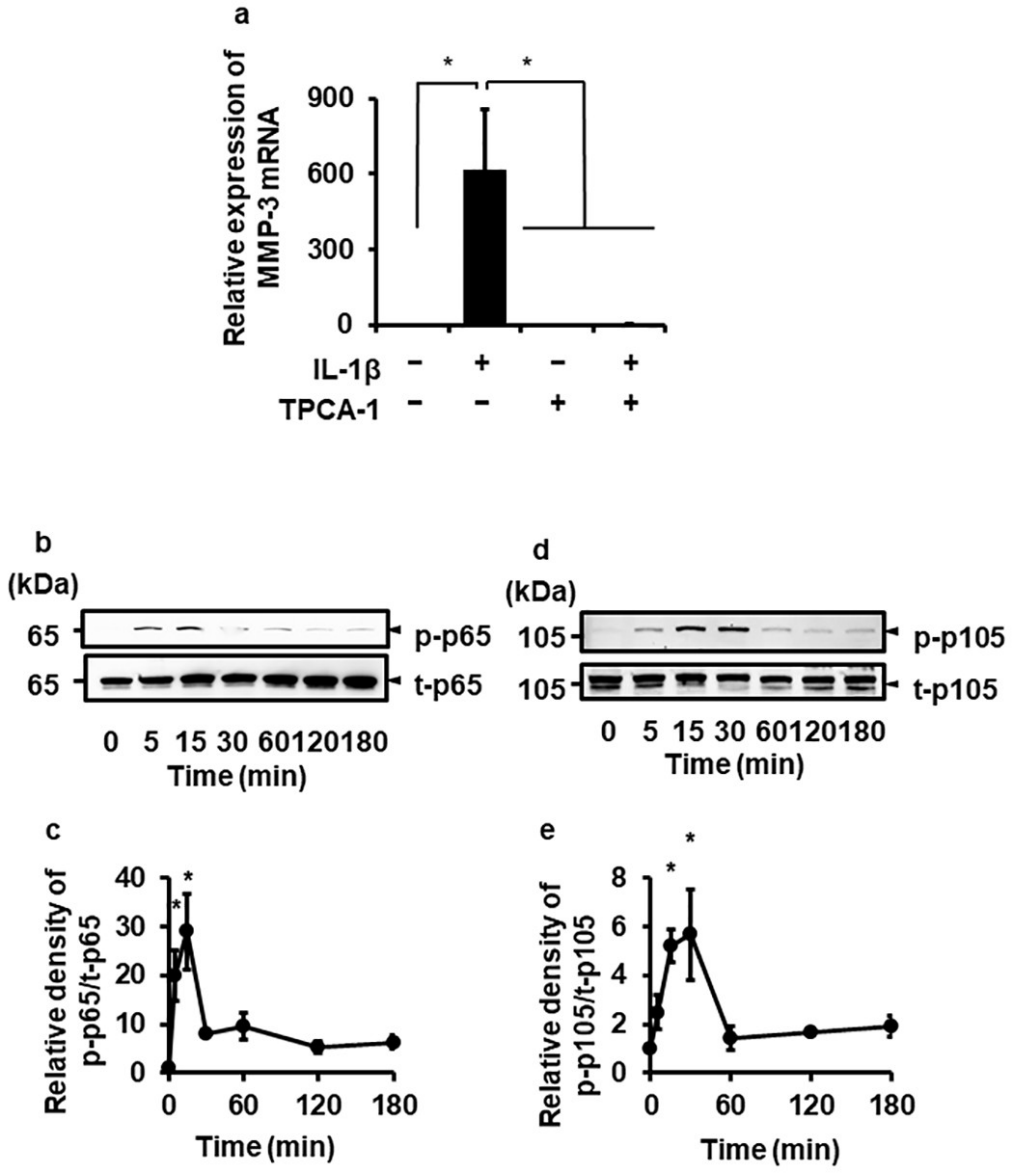
The involvement of NF-κB activation in IL-1β-mediated-MMP-3 expression. (a) The effect of IL-1β (100 pM, 24 h) on MMP-3 mRNA expression in melanoma cells with or without the NF-κB inhibitor, TPCA-1 (10 μM, 1 h). TBP was used as a reference, and the relative expression levels of MMP-3 mRNA in IL-1β-stimulated melanoma cells were compared with that of 0 h. The results are shown as mean ± standard error (SE) of biological triplicates. *P<0.05. (b-e) IL-1β-mediated activation of NF-κB. Phosphorylated p65/RelA (p-p65) and p105 (p-p105), and total p65/RelA (t-p65) and p105 (t-p105) were detected by Western blotting. (b, d) Representative images of the time-dependent changes of phosphorylated p65/RelA (p-p65), total p65/RelA (t-p65), phosphorylated p105 (p-p105), and total p105 (t-p105) with IL-1β stimulation (100 pM). The phosphorylation levels of p65/RelA and p105 were transiently increased. Relative changes of p-p65/t-p65 (c) and p-p105/t-p105 (d) compared to those at 0 h. The results are shown as mean ± SE of biological triplicates. *P<0.05.

IL-1β induces the NF-κB activation, which upregulates the expression of many targets (*i*.*e*., MMPs and inflammatory cytokines) at the transcriptional level. Thus, we investigated whether the inhibition of NF-κB attenuated MMP-3 mRNA expression through a pharmacological approach. As shown in Fig 2a, the significant inhibition in IL-1β-mediated-MMP-3 mRNA expression was observed in the cells pretreated with TPCA-1 (10 μM) for 1 h, suggesting that the activation of NF-κB is involved in MMP-3 mRNA expression in IL-1β-stimulated canine melanoma cells.

**Fig 3:**
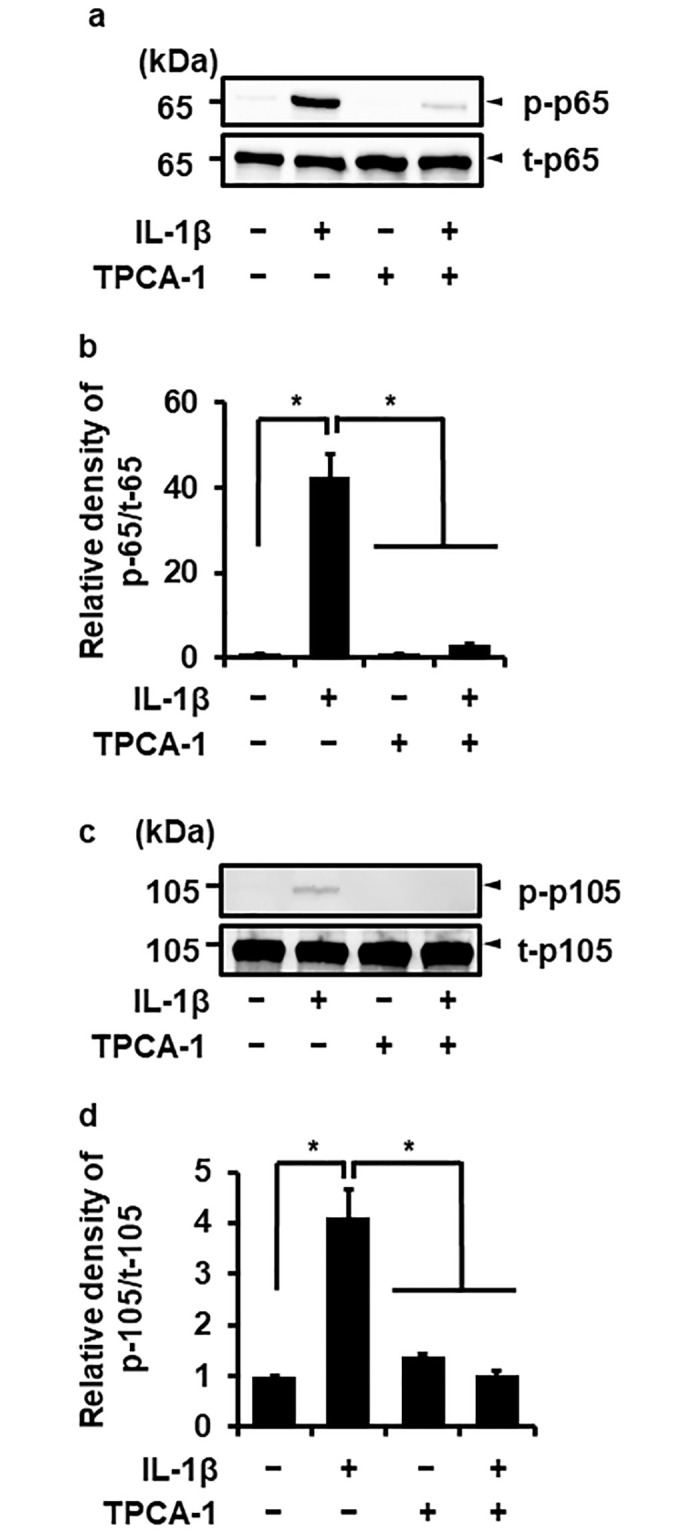
Effect of the NF-κB inhibitor on the IL-1β-induced phosphorylation of p65/RelA and p105. The cells were pretreated with the NF-κB inhibitor, TPCA-1 (10 μM, 1 h), followed by the stimulation of IL-1β (100 pM) for 15 min. Phosphorylated p65/RelA (p-p65) and p105 (p-p105), and total p65/RelA (t-p65) and p105 (t-p105) were detected by Western blotting. Representative images of the inhibitory effect of TPCA-1 on IL-1β-induced phosphorylation of p65/RelA (a) and p105 (c) are shown. The relative levels of [p-p65]/[t-p65] (b) and [p-p-105]/[t-105] (d) relative to levels without the inhibitor and IL-1β are illustrated. The results are represented as mean ± SE of biological triplicates. *P<0.05.

We confirmed whether IL-1β induces the canonical NF-κB activation in canine melanoma cells by detecting the phosphorylation of p65/RelA and p105. When melanoma cells were treated with IL-1β (100 pM) for 0–180 min, p65/RelA (Fig 2b and 2c) and p105 phosphorylation (Fig 2d and 2e) was observed at 5–15 min and 5–30 min after stimulation, respectively. Such p65/RelA and p105 phosphorylation induced by IL-1β was reduced in melanoma cells pretreated with TPCA-1 (Fig 3a–3d). Together, these findings strongly suggest that IL-1β mediates MMP-3 mRNA expression via NF-κB activation.

### p65/RelA dominantly contributes to IL-1β-provoked MMP-3 mRNA expression

**Fig 4:**
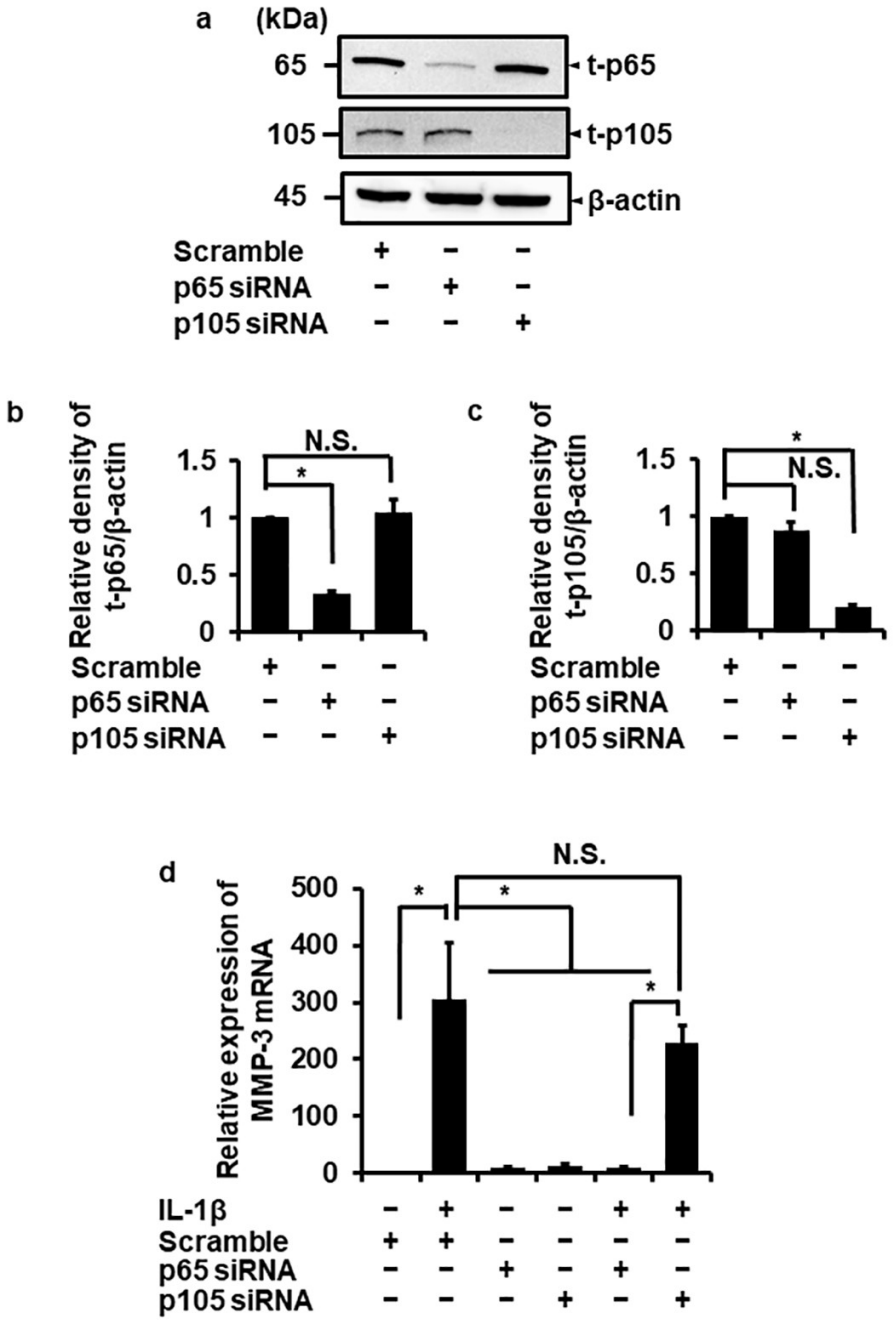
Attenuating IL-1β-induced MMP-3 mRNA expression in melanoma cells transfected with p65/RelA siRNA, but not with p105 siRNA. (a-c) The effect of the depletion of t-p65/RelA, t-105, and β-actin in melanoma cells transfected with p65/RelA, p105, or scrambled siRNAs. Representative images of Western blotting (a) and relative density of the expression of t-p65/RelA (b) or t-p105 (c) in each siRNA-transfected cell compared with those in the cells transfected with scramble siRNA. Depletion of p65/RelA or p105 was observed in melanoma cells transfected with p65/RelA or p105 siRNA, respectively, but not in the cells transfected with scrambled siRNA. β-actin was used as a reference. (d) The effect of siRNA transfection for p65/RelA or p105 on the MMP-3 expression in IL-1β-treated cells. Melanoma cells transfected with p65/RelA, p105, or scrambled siRNAs following the stimulation of IL-1β (100 pM) for 24 h. Relative expression of MMP-3 mRNA was evaluated. TBP was used as a reference. The attenuation of MMP-3 expression was observed in p65/RelA-depleted cells but not in the cells transfected with p105 siRNA or scrambled siRNA. The results are represented as mean ± standard error (SE) of biological triplicates. *P<0.05.

To confirm the contribution of NF-κB to MMP-3 mRNA expression mediated by IL-1β, we investigated whether IL-1β induced the mRNA expression of MMP-3 in p65/RelA or p105 knockdown melanoma cells. We confirmed that the protein expression of p65/RelA or p105 was significantly inhibited in the cells transfected with siRNA for p65/RelA or p105, respectively (Fig 4a–4c).

**Fig 5:**
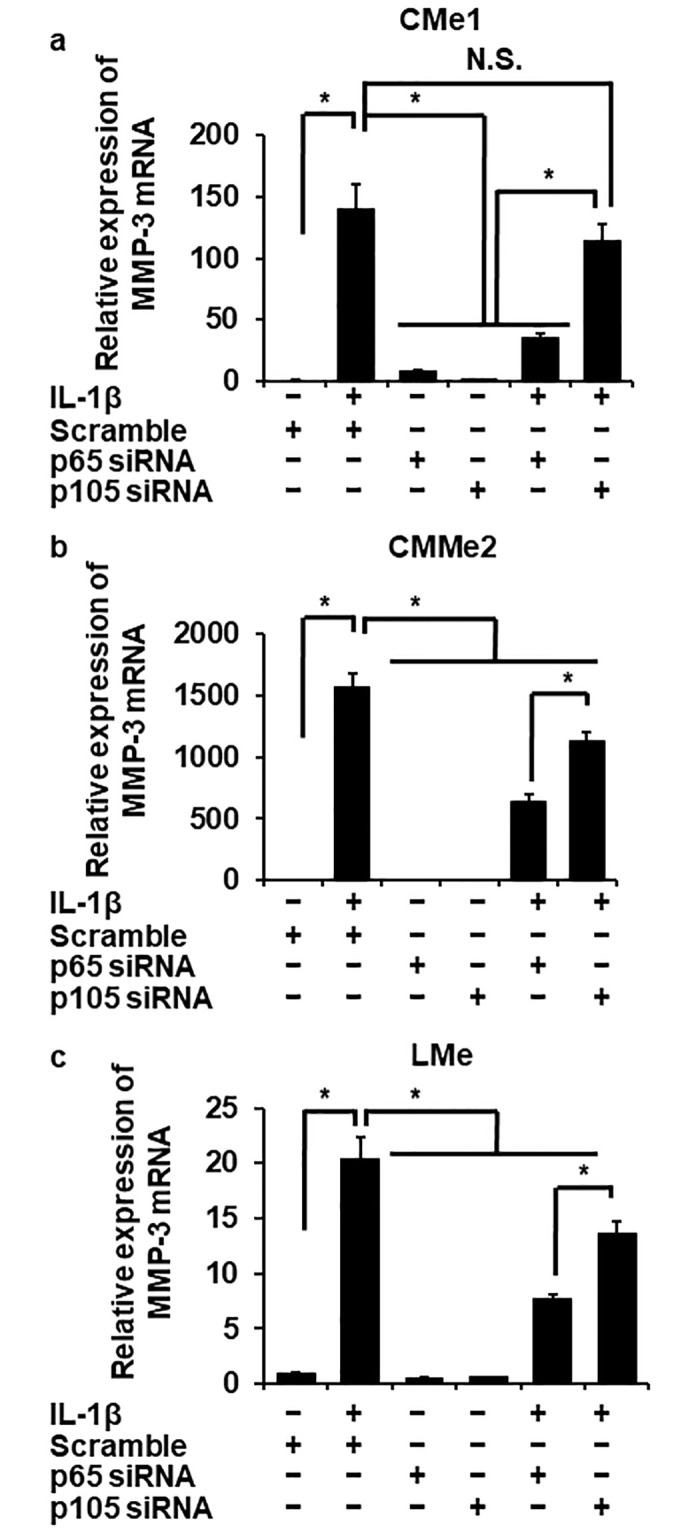
Attenuation of IL-1β-induced MMP-3 mRNA expression in other sets of melanoma cells transfected with p65/RelA siRNA, but not with p105 siRNA. Melanoma cells (a, CMe1; b, eCMMe2; c, LMe) transfected with p65/RelA, p105, or scrambled siRNAs following the stimulation of IL-1β (100 pM) for 24 h. Relative expression of MMP-3 mRNA was evaluated. TBP was used as a reference. The attenuation of MMP-3 expression was observed in p65/RelA-depleted cells but not in the cells transfected with p105 siRNA or scrambled siRNA. The results are represented as mean ± standard error (SE) of biological triplicates. *P<0.05.

Then we evaluated the effect of IL-1β on MMP-3 mRNA expression in p65/RelA- or p105-depleted melanoma cells. Interestingly, we observed the significant attenuation in MMP-3 mRNA expression in p65/RelA-depleted melanoma cells compared to p105 siRNA or the control scramble siRNA-transfected cells (Figs 4d and 5). These results strongly suggest that NF-κB p65/RelA, but not p105, plays a crucial role in MMP-3 expression in IL-1β-treated canine melanoma cells.

## Discussion

In the present study, we demonstrated that IL-1β triggers MMP-3 expression and release in canine melanoma cells. MMP-3 is a metalloproteinase that cleaves several ECM components, including collagens, laminins, and proteoglycans [15, 16], and pro-MMPs, such as MMP-1, MMP-9, and MMP-13, for their activation [16–19].

The multiple roles of MMPs have been reported; the promotion of cellular migration, angiogenesis, and TME creation. Previously, the role of MMPs in cancer pathogenesis hypothesized that MMPs mediate the degradation of ECM and make tunnels, which enables cancer cells to invade. The tunnel spaces also allow endothelial cells to migrate and form new blood vessels. In addition to the typical action of MMPs in ECM, the involvement of the cleavage in cellular components (i.e., membrane receptors and precursor for enzymes) has been reported. The cleavage of proteins by MMPs uncovers the cryptic domains, which mediates the activation and interaction of target proteins. Thus, the regulation of expression and activation of MMPs has been considered a promising anti-tumor therapy approach. As MMP-3 expression is observed in various types of malignant cells [16], including human malignant melanoma, MMP-3 has been considered to play a role in malignant processes [23, 24]. High expression levels of MMP-3 in human patients have been reported to be correlated with shorter disease-free survival in human metastatic melanoma [57]. The correlation between high expression of MMP-3 and the survival of patients has also been demonstrated by TCGA [58]. In rat embryo cell lines, high levels of MMP-3 expression were found to be associated with the metastatic potential of transformed cells [59]. MMP-3 protein expression and release in human melanoma have been observed in aggressive and highly metastatic cell lines [58, 60]. Experimental metastasis and cell growth were significantly increased in nude mice injected with human melanoma cells overexpressing MMP-3 but were inhibited in nude mice injected with MMP-3-silenced melanoma cells [58]. In our study, an MMP-3 inhibitor significantly inhibited IL-1β-mediated melanoma cell migration, supporting the role of MMP-3 in melanoma metastasis.

IL-1β was demonstrated to mediate MMP-3 expression and release in melanoma cells. In a previous study, plasma levels of MMP-3 were not found to differ between patients with melanoma and healthy controls [61, 62]. IL-1β is a key mediator of inflammation and immunity, contributing to tumor invasiveness and metastasis via the expression of various genes for arranging the tumor microenvironment [8–11]. In this study, released MMP-3 was found to mediate the migration of melanoma cells in an autocrine manner. MMP-3 deficiency in melanoma is reported to reduce the formation of 3D-tumoroids in vitro, whereas the addition of MMP-3-enriched extracellular vesicles promotes the tumorigenicity and proliferation of MMP-3 (-/-) melanoma cells [63]. Therefore, IL-1β-mediated-MMP-3 may contribute to malignant tumor processes by creating a suitable microenvironment for tumor development and progression.

In the present study, we showed that NF-κB p65/RelA contributes to MMP-3 expression induced by IL-1β in melanoma cells. NF-κB p65/RelA subunit exists as a homodimer or heterodimer with the p50 subunit, which forms a complex with inhibitory IκB proteins, such as IκBα, and localizes in the cytoplasm in unstimulated cells [64–68]. After stimulation, such as with the cytokine IL-1β, the IκB protein is phosphorylated by IκB kinase, which is followed by protein ubiquitination and degradation. Consequently, dimeric forms of NF-κB released from the inhibitory protein translocate to the nucleus to control gene expression [25, 29, 30, 69]. Such a signaling process is referred to as the canonical NF-κB signaling pathway. In our study, treatment with the IκB kinase inhibitor TPCA-1 resulted in the inhibition of IL-1β-induced MMP-3 mRNA expression and protein release in melanoma cells. This result suggests the involvement of the activation of IκB kinases in IL-1β-induced MMP-3 mRNA expression in melanoma cells.

Although the transcriptional regulatory elements in the MMP-3 gene by NF-κB have not been identified, NF-κB has been reported to contribute to the transcriptional regulation of human MMP-3 expression [70–75]. As a polymorphism in the promoter of the MMP-3 gene influences its transcription, the polymorphic site 5A and 6A alleles have been suggested to interact with NF-κB [74–77]. Recombinant p50 homodimers were reported to bind to the polymorphic sites of the MMP-3 gene, and overexpression of p50 and p65/RelA in fibroblasts resulted in the repression of MMP-3 expression [74, 77]. However, p50 has no transcriptional activation domains. Therefore, p50 was considered a repressor of MMP-3 expression [74, 77, 78]. However, the overexpression of p50 and p65/RelA resulted in the activation of MMP-3 expression via monocyte polymorphism sites [75]. In our study, p105 and p65/RelA were found to be activated. However, IL-1β failed to mediate the expression of MMP-3 mRNA in p65/RelA-knockdown melanoma cells but not in p105-knockdown cells. Therefore, the mechanism of p65/RelA-dependent MMP-3 expression may be distinct from such a scenario. Currently, p65/RelA dimerization and DNA-binding have been demonstrated to be important for the induction of gene expression in cells stimulated with cytokines, such as TNF-α, using p65/RelA-deficient HeLa cells and mouse embryonic fibroblasts [77]. In the study, cooperation with other transcription factors (e.g., AP-1) has been suggested to contribute to gene expression via p65/RelA-mediated transcription [78]. Transcription factors, such as NFAT1 [77], zinc-binding protein-89 [79, 80], SOX2 [81], and actin-binding protein α-actinin 4 [82], have been demonstrated to be involved in MMP-3 expression. Therefore, the relationship between p65/RelA and such transcription factors must be determined to elucidate the expression of MMP-3 via activation of NF-κB p65/RelA in canine melanoma cells.

## Conclusion

In conclusion, we demonstrated that IL-1β induces the activation of NF-κB p65/RelA, but not p105, which contributes to IL-1β-induced MMP-3 expression in canine melanoma cells. Previously, we observed that p65/RelA and p105 activation are necessary for COX-2 expression mediated by IL-1β in canine melanoma cells (39). Therefore, understanding the precise function of each NF-κB subunit for IL-1β-evoked gene expression is important for developing new therapeutic strategies for melanoma.

## Supporting information

S1 Fig The effect of IL-1β on intracellular pH (a) and the mRNA expression of NHE1 (b). The cells were treated with (closed) or without (open) 100 pM IL-1β. IL-1β failed to induce the changes in intracellular pH (a, n = 20 cells, randomly selected ×20 fields from triplicate samples) and mRNA expression of NHE1 (b). Data are shown as the mean ± standard error of three independent experiments.
(PDF)

S2 Fig The effect of IL-1β on cellular invasion (a) and adhesion (b). a, In melanoma cells, cellular invasion was undetectable. b, Cellular adhesion of IL-1β, UK356618, and UK356618+ IL-1β-treated cells showed no significant difference compared to control. Data are shown as the mean ± standard error of three independent experiments.
(PDF)

S1 Raw images (PDF)
