# Supplementary material for: Interleukin-1β triggers matrix metalloprotease-3 expression through p65/RelA activation in melanoma cells
Source: PLoS One. 2022 Nov 29;17(11):e0278220. doi: 10.1371/journal.pone.0278220 (PMC9707762; doi:10.1371/journal.pone.0278220)
Supplement: S1 Fig — The cells were treated with (closed) or without (open) 100 pM IL-1β. IL-1β failed to induce the changes in intracellular pH (a, n = 20 cells, randomly selected ×20 fields from triplicate samples) and mRNA expression of NHE1 (b). Data are shown as the mean ± standard error of three independent experiments. (PDF) [file pone.0278220.s001.pdf]

a

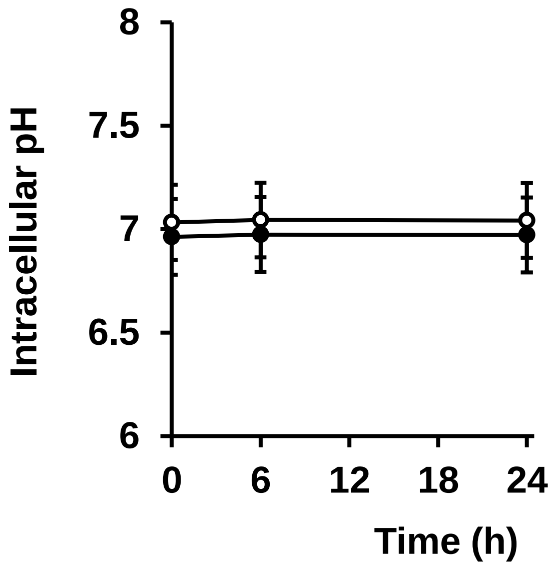

b

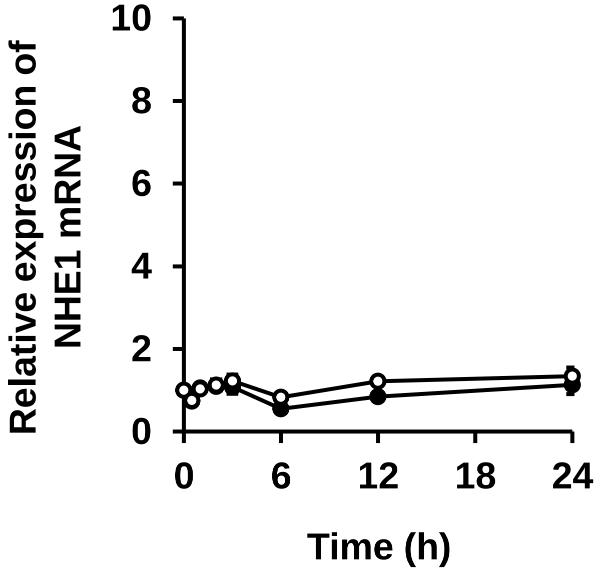

S1 Fig. The effect of IL-1β on intracellular pH (a) and the mRNA expression of NHE1 (b). The cells were treated with (closed) or without (open) 100 pM IL-1β. IL-1β failed to induce the changes in intracellular pH (a, n = 20 cells, randomly selected ×20 fields from triplicate samples) and mRNA expression of NHE1 (b). Data are shown as the mean ± standard error of three independent experiments.
