# Supplementary material for: Interleukin-1β triggers matrix metalloprotease-3 expression through p65/RelA activation in melanoma cells
Source: PLoS One. 2022 Nov 29;17(11):e0278220. doi: 10.1371/journal.pone.0278220 (PMC9707762; doi:10.1371/journal.pone.0278220)
Supplement: S2 Fig — a, In melanoma cells, cellular invasion was undetectable. b, Cellular adhesion of IL-1β, UK356618, and UK356618+ IL-1β-treated cells showed no significant difference compared to control. Data are shown as the mean ± standard error of three independent experiments. (PDF) [file pone.0278220.s002.pdf]

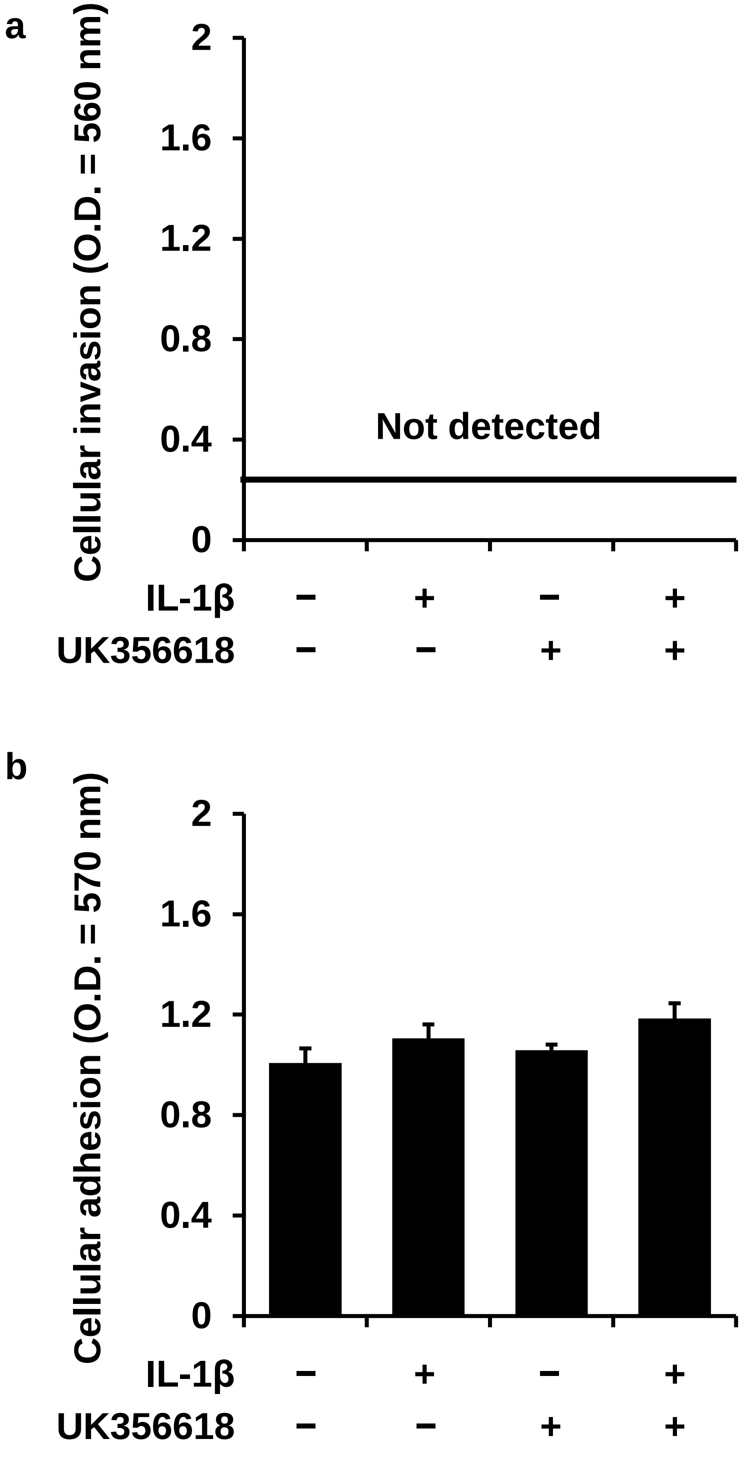

S2 Fig. The effect of IL-1β on cellular invasion (a) and adhesion (b). a, In melanoma cells, cellular invasion was undetectable. b, Cellular adhesion of IL-1β, UK356618, and UK356618+ IL-1β-treated cells showed no significant difference compared to control. Data are shown as the mean ± standard error of three independent experiments.
