# Supplementary figures and images for: Interleukin-1β triggers matrix metalloprotease-3 expression through p65/RelA activation in melanoma cells
Source: PLoS One. 2022 Nov 29;17(11):e0278220. doi: 10.1371/journal.pone.0278220 (PMC9707762; doi:10.1371/journal.pone.0278220)

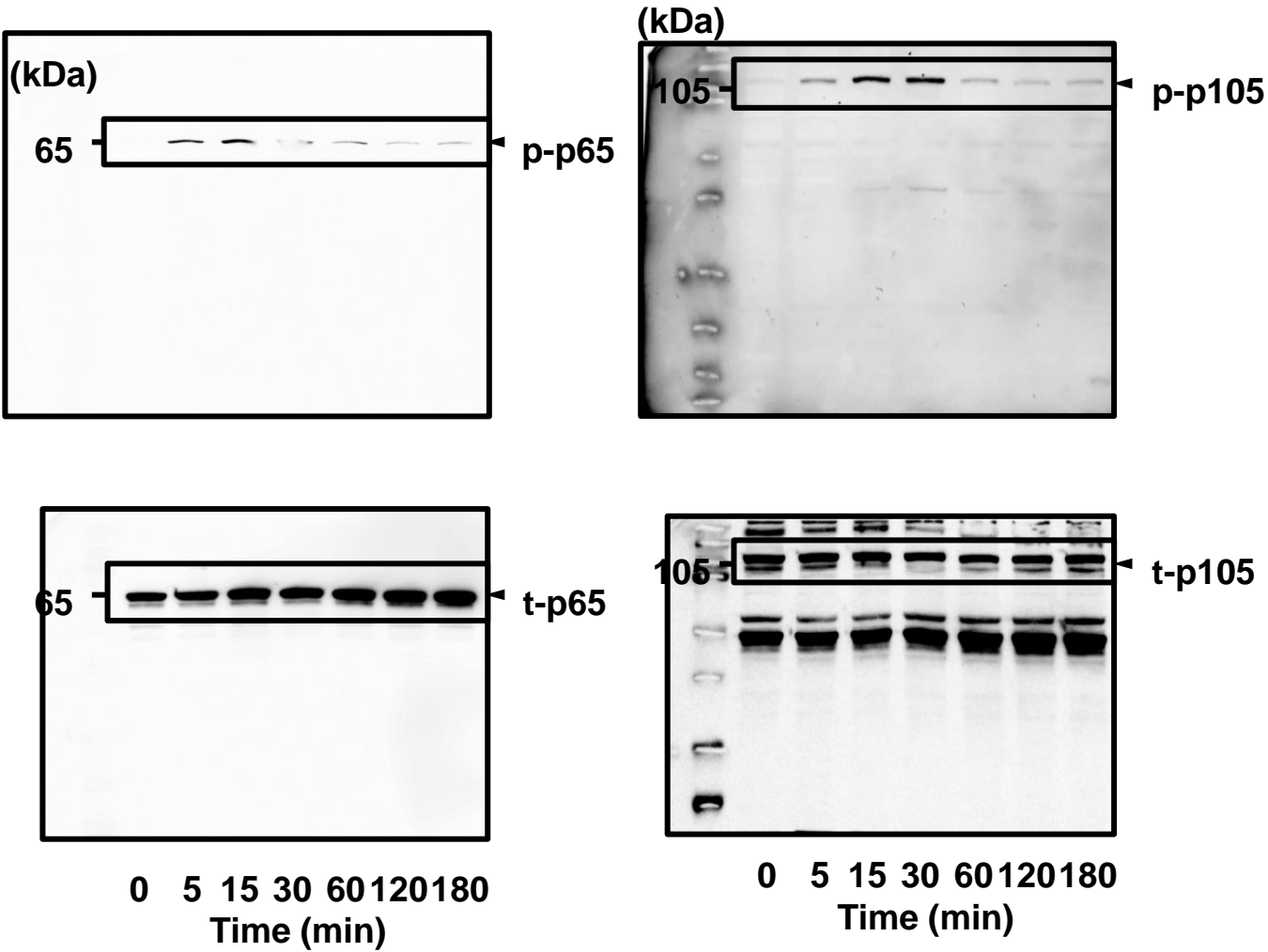

Uncropped images for the blots shown in Fig. 2b and d

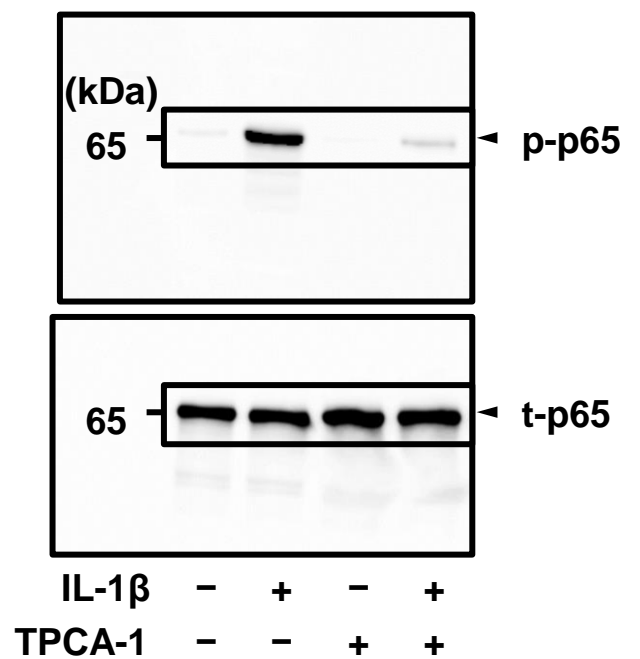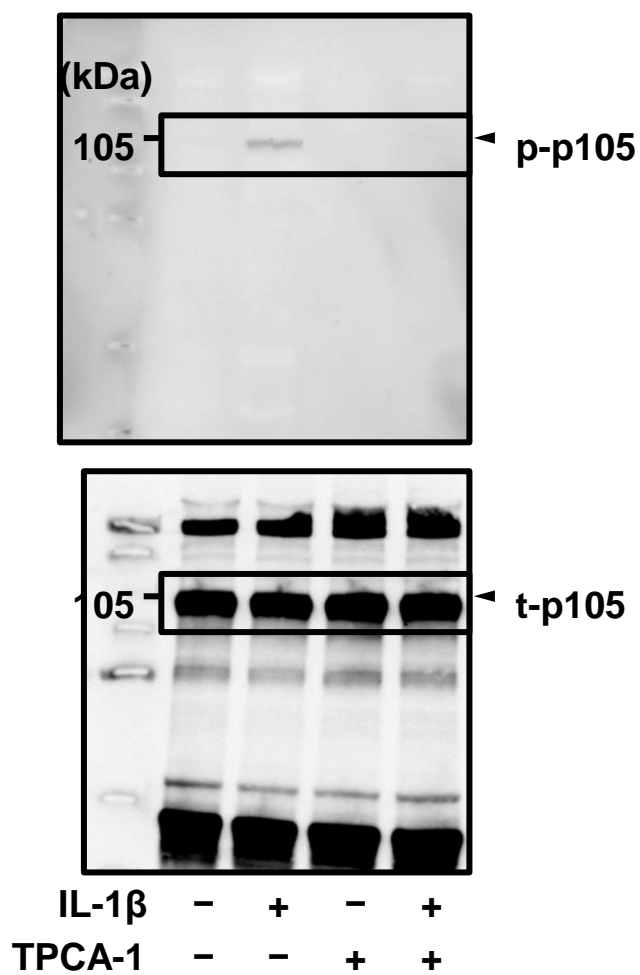

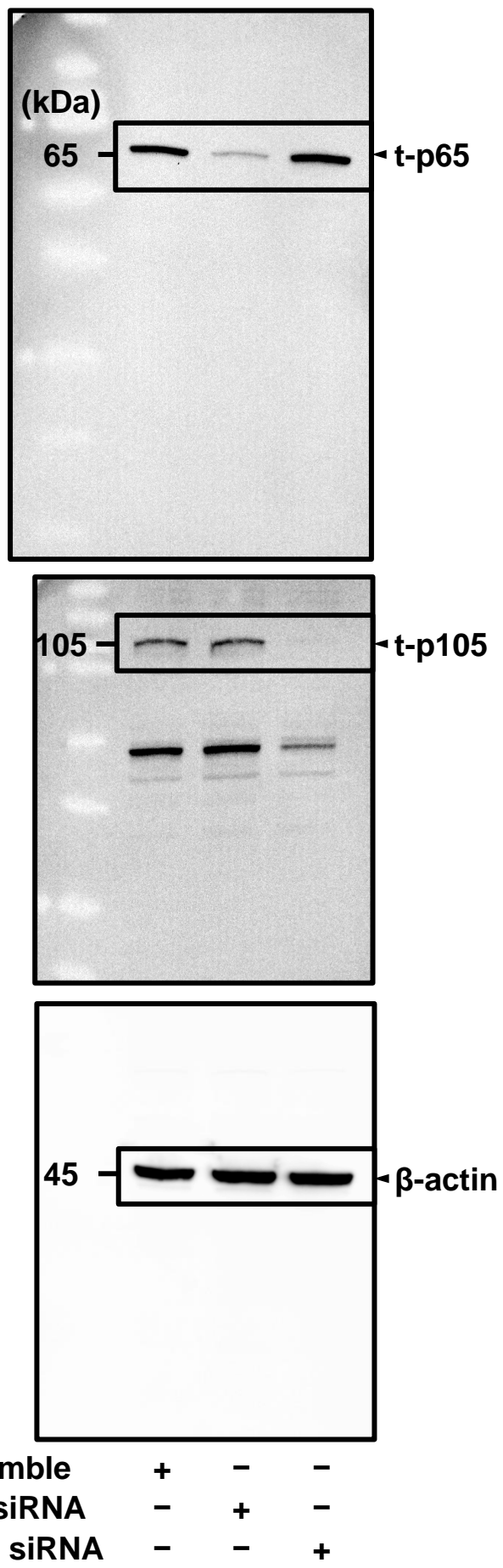

Uncropped images for the blots shown in Fig. 4a

Supplement: S1 Raw images — (PDF) [file pone.0278220.s003.pdf]
